# (*Z*)-*N*-(2-Chloro­benzyl­idene)aniline *N*-oxide

**DOI:** 10.1107/S1600536811015923

**Published:** 2011-05-07

**Authors:** Ying Fu, Yanhua Liu, Yanshou Yang, Yaojuan Chen

**Affiliations:** a Key Laboratory of Polymer Materials of Gansu Province, College of Chemistry and Chemical Engineering, Northwest Normal University, Lanzhou, An’ning East Road No. 967, Gansu Province 730070, People’s Republic of China

## Abstract

In the title compound, C_13_H_10_ClNO, the central C—N bond has considerable double-bond character and the N—O bond indicates a formal negative charge on the O atom. The mol­ecule is stabilized by an intra­molecular C—H⋯O hydrogen bond. The geometry about the C=N bond is *Z* [C—C—N—O torsion angle = −4.2 (3)°] and the phenyl and benzene rings are *trans*-oriented around the C=N bond. The phenyl and benzene rings make a dihedral angle of 56.99 (2)°.

## Related literature

For the crystal structures of diphenyl nitrone derivatives, see: Vijayalakshmi *et al.* (1997 ▶, 2000 ▶); Kang *et al.* (2000 ▶); Bedford *et al.* (1991 ▶); Mothi Mohamed *et al.* (2003 ▶); Brown & Trefonas (1973 ▶); Chandrasekar & Panchanatheswaran (2000 ▶). 
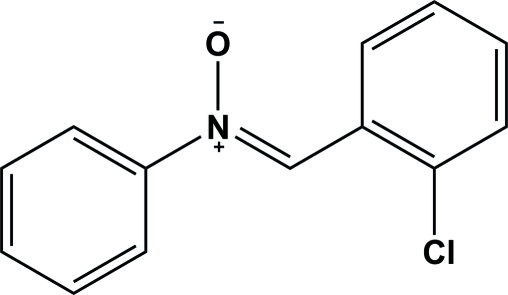


## Experimental

### 

#### Crystal data

C_13_H_10_ClNO
*M* *_r_* = 231.67
Monoclinic, 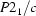

*a* = 9.7302 (3) Å
*b* = 9.7389 (3) Å
*c* = 11.7460 (3) Å
β = 104.093 (1)°
*V* = 1079.57 (5) Å^3^
*Z* = 4
Mo *K*α radiation
μ = 0.33 mm^−1^
*T* = 296 K
0.45 × 0.42 × 0.41 mm

#### Data collection

Bruker APEXII CCD diffractometer
Absorption correction: multi-scan (*SADABS*; Sheldrick, 2008*a* ▶) *T*_min_ = 0.866, *T*_max_ = 0.877
6083 measured reflections
2232 independent reflections
1904 reflections with *I* > 2σ(*I*)
*R*_int_ = 0.027

#### Refinement

*R*[*F* ^2^ > 2σ(*F* ^2^)] = 0.036
*wR*(*F* ^2^) = 0.136
*S* = 1.05
2232 reflections
145 parameters
H-atom parameters constrained
Δρ_max_ = 0.22 e Å^−3^
Δρ_min_ = −0.28 e Å^−3^

### 

Data collection: *APEX2* (Bruker, 2008 ▶); cell refinement: *SAINT* (Bruker, 2008 ▶); data reduction: *SAINT*; program(s) used to solve structure: *SHELXS97* (Sheldrick, 2008*b* ▶); program(s) used to refine structure: *SHELXL97* (Sheldrick, 2008*b* ▶); molecular graphics: *SHELXTL*; software used to prepare material for publication: *SHELXTL*.

## Supplementary Material

Crystal structure: contains datablocks I, global. DOI: 10.1107/S1600536811015923/bx2346sup1.cif

Structure factors: contains datablocks I. DOI: 10.1107/S1600536811015923/bx2346Isup2.hkl

Supplementary material file. DOI: 10.1107/S1600536811015923/bx2346Isup3.cml

Additional supplementary materials:  crystallographic information; 3D view; checkCIF report

## Figures and Tables

**Table 1:** Hydrogen-bond geometry (Å, °)

*D*—H⋯*A*	*D*—H	H⋯*A*	*D*⋯*A*	*D*—H⋯*A*
C5—H5⋯O1	0.93	2.26	2.857 (2)	121

**Fig. 1.**
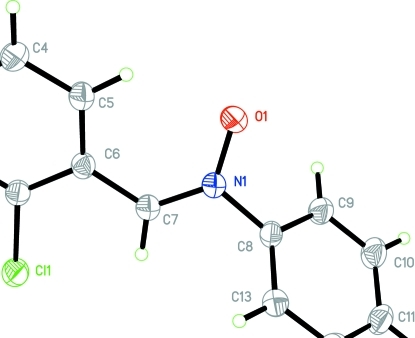
The molecular structure of (I), with atom labels and 30% probability displacement ellipsoids.

## supplementary crystallographic information

### Comment

In the crystal structure of the title compound, (I) the bond lenghts C=N and N—O are 1.304 (2) and 1.2933 (16) Å respectively which are similar to the values observed in other nitrones compounds (Vijayalakshmi, *et al.*, 1997 and 2000). The central moiety is planar as seen in the C6—C7—N1—C8 torsion angle of 178.20 (14)° . The phenyl and benzene substituents are twisted out of this plane by 47.4 (2) ) and 14.3( 2)° respectively and are oriented trans conformation respect to the C=N bond. Two  short intermolecular contact are observed, Cl···H3^i^ 2.884 Å and C12···C6^i^ 3.382 (2)Å [symmetry code: (i) x, 1.5-y, -1/2+z].

### Experimental

A solution of 2-chlorobenzaldehyde (2.80 g) was added dropwise with stirring to a solution of *N*-phenylhydroxylamine (2.1 g) in ethanol (20 ml). The mixture was heated for about 1 h at 333 K. The crystals were crystallized from ethanol [m.p. 426 K; yield 1.90 g (82%)].

### Refinement

H atoms bonded to C atoms were located in a difference map and refined with distance restraints  and refined using a riding model  with C—H = 0.93Å and with *U*_iso_(H) = 1.2  times *U*_eq_(C).

### Crystal data

**Table:**

C_13_H_10_ClNO	*F*(000) = 480
*M**_r_* = 231.67	*D*_x_ = 1.425 Mg m^−^^3^
Monoclinic, *P*2_1_/*c*	Melting point: 362 K
Hall symbol: -P 2ybc	Mo *K*α radiation, λ = 0.71073 Å
*a* = 9.7302 (3) Å	Cell parameters from 2703 reflections
*b* = 9.7389 (3) Å	θ = 2.8–29.3°
*c* = 11.7460 (3) Å	µ = 0.33 mm^−^^1^
β = 104.093 (1)°	*T* = 296 K
*V* = 1079.57 (5) Å^3^	Block, yellow
*Z* = 4	0.45 × 0.42 × 0.41 mm

### Data collection

**Table:**

Bruker APEXII CCD diffractometer	2232 independent reflections
Radiation source: fine-focus sealed tube	1904 reflections with *I* > 2σ(*I*)
graphite	*R*_int_ = 0.027
φ and ω scans	θ_max_ = 26.5°, θ_min_ = 2.2°
Absorption correction: multi-scan (*SADABS*; Sheldrick, 2008*a*)	*h* = −12→12
*T*_min_ = 0.866, *T*_max_ = 0.877	*k* = −11→12
6083 measured reflections	*l* = −14→14

### Refinement

**Table:**

Refinement on *F*^2^	Primary atom site location: structure-invariant direct methods
Least-squares matrix: full	Secondary atom site location: difference Fourier map
*R*[*F*^2^ > 2σ(*F*^2^)] = 0.036	Hydrogen site location: inferred from neighbouring sites
*wR*(*F*^2^) = 0.136	H-atom parameters constrained
*S* = 1.05	*w* = 1/[σ^2^(*F*_o_^2^) + (0.1*P*)^2^] where *P* = (*F*_o_^2^ + 2*F*_c_^2^)/3
2232 reflections	(Δ/σ)_max_ = 0.001
145 parameters	Δρ_max_ = 0.22 e Å^−^^3^
0 restraints	Δρ_min_ = −0.28 e Å^−^^3^

### Special details

**Table:**

Geometry. All e.s.d.'s (except the e.s.d. in the dihedral angle between two l.s. planes) are estimated using the full covariance matrix. The cell e.s.d.'s are taken into account individually in the estimation of e.s.d.'s in distances, angles and torsion angles; correlations between e.s.d.'s in cell parameters are only used when they are defined by crystal symmetry. An approximate (isotropic) treatment of cell e.s.d.'s is used for estimating e.s.d.'s involving l.s. planes.
Refinement. Refinement of *F*^2^ against ALL reflections. The weighted *R*-factor *wR* and goodness of fit *S* are based on *F*^2^, conventional *R*-factors *R* are based on *F*, with *F* set to zero for negative *F*^2^. The threshold expression of *F*^2^ > σ(*F*^2^) is used only for calculating *R*-factors(gt) *etc*. and is not relevant to the choice of reflections for refinement. *R*-factors based on *F*^2^ are statistically about twice as large as those based on *F*, and *R*- factors based on ALL data will be even larger.

### Fractional atomic coordinates and isotropic or equivalent isotropic displacement parameters (Å^2^)

**Table:**

	*x*	*y*	*z*	*U*_iso_*/*U*_eq_	
C1	0.59092 (15)	0.85392 (16)	1.09921 (12)	0.0398 (4)	
C2	0.55547 (18)	0.86628 (18)	1.20546 (14)	0.0493 (4)	
H2	0.4895	0.8070	1.2245	0.059*	
C3	0.6185 (2)	0.96710 (19)	1.28344 (15)	0.0544 (4)	
H3	0.5941	0.9766	1.3548	0.065*	
C4	0.7169 (2)	1.05308 (19)	1.25583 (14)	0.0558 (5)	
H4	0.7610	1.1192	1.3095	0.067*	
C5	0.75133 (19)	1.04225 (18)	1.14841 (13)	0.0482 (4)	
H5	0.8172	1.1024	1.1304	0.058*	
C6	0.68853 (15)	0.94220 (16)	1.06663 (12)	0.0387 (3)	
C7	0.72415 (16)	0.92349 (15)	0.95480 (13)	0.0409 (4)	
H7	0.6940	0.8423	0.9147	0.049*	
C8	0.82470 (16)	0.97275 (16)	0.79355 (13)	0.0401 (4)	
C9	0.80082 (18)	1.07137 (17)	0.70579 (14)	0.0490 (4)	
H9	0.7639	1.1567	0.7181	0.059*	
C10	0.8323 (2)	1.0418 (2)	0.60028 (15)	0.0566 (5)	
H10	0.8157	1.1068	0.5404	0.068*	
C11	0.88845 (18)	0.9152 (2)	0.58376 (15)	0.0546 (5)	
H11	0.9096	0.8953	0.5125	0.066*	
C12	0.91332 (18)	0.8187 (2)	0.67115 (16)	0.0523 (4)	
H12	0.9519	0.7342	0.6591	0.063*	
C13	0.88125 (16)	0.84651 (17)	0.77743 (14)	0.0465 (4)	
H13	0.8976	0.7811	0.8369	0.056*	
Cl1	0.51025 (5)	0.72300 (5)	1.00632 (4)	0.0567 (2)	
N1	0.79421 (14)	1.00909 (12)	0.90471 (11)	0.0434 (3)	
O1	0.83697 (18)	1.12972 (13)	0.94397 (11)	0.0729 (5)	

### Atomic displacement parameters (Å^2^)

**Table:**

	*U*^11^	*U*^22^	*U*^33^	*U*^12^	*U*^13^	*U*^23^
C1	0.0408 (7)	0.0398 (8)	0.0400 (8)	0.0037 (6)	0.0119 (6)	0.0026 (6)
C2	0.0545 (9)	0.0499 (9)	0.0490 (9)	0.0006 (7)	0.0230 (7)	0.0042 (7)
C3	0.0740 (11)	0.0569 (10)	0.0386 (8)	0.0025 (9)	0.0254 (8)	0.0026 (8)
C4	0.0775 (12)	0.0533 (10)	0.0367 (8)	−0.0070 (9)	0.0140 (8)	−0.0028 (8)
C5	0.0608 (10)	0.0469 (9)	0.0389 (8)	−0.0080 (8)	0.0161 (7)	0.0020 (7)
C6	0.0421 (7)	0.0386 (8)	0.0361 (7)	0.0033 (6)	0.0111 (6)	0.0049 (6)
C7	0.0458 (8)	0.0403 (8)	0.0383 (7)	−0.0029 (6)	0.0132 (6)	0.0007 (6)
C8	0.0415 (8)	0.0437 (8)	0.0381 (8)	−0.0054 (6)	0.0152 (6)	−0.0007 (6)
C9	0.0598 (10)	0.0455 (9)	0.0457 (9)	0.0042 (7)	0.0207 (8)	0.0052 (7)
C10	0.0655 (11)	0.0657 (11)	0.0422 (9)	−0.0025 (9)	0.0196 (8)	0.0061 (8)
C11	0.0540 (10)	0.0695 (12)	0.0454 (9)	−0.0114 (8)	0.0219 (7)	−0.0129 (8)
C12	0.0464 (9)	0.0498 (9)	0.0644 (11)	−0.0030 (7)	0.0208 (8)	−0.0153 (8)
C13	0.0477 (8)	0.0439 (8)	0.0506 (9)	−0.0010 (7)	0.0175 (7)	0.0016 (7)
Cl1	0.0618 (3)	0.0572 (3)	0.0559 (3)	−0.01765 (19)	0.0235 (2)	−0.01032 (18)
N1	0.0543 (8)	0.0394 (7)	0.0406 (7)	−0.0058 (5)	0.0192 (6)	−0.0017 (5)
O1	0.1234 (12)	0.0490 (8)	0.0603 (8)	−0.0311 (8)	0.0496 (8)	−0.0134 (6)

### Geometric parameters (Å, °)

**Table:**

C1—C2	1.379 (2)	C8—C13	1.379 (2)
C1—C6	1.402 (2)	C8—C9	1.387 (2)
C1—Cl1	1.7364 (16)	C8—N1	1.451 (2)
C2—C3	1.380 (3)	C9—C10	1.377 (2)
C2—H2	0.9300	C9—H9	0.9300
C3—C4	1.369 (3)	C10—C11	1.381 (3)
C3—H3	0.9300	C10—H10	0.9300
C4—C5	1.386 (2)	C11—C12	1.369 (3)
C4—H4	0.9300	C11—H11	0.9300
C5—C6	1.400 (2)	C12—C13	1.385 (2)
C5—H5	0.9300	C12—H12	0.9300
C6—C7	1.449 (2)	C13—H13	0.9300
C7—N1	1.304 (2)	N1—O1	1.2933 (16)
C7—H7	0.9300		
			
C2—C1—C6	121.95 (14)	C13—C8—C9	121.05 (15)
C2—C1—Cl1	117.28 (12)	C13—C8—N1	121.12 (13)
C6—C1—Cl1	120.77 (11)	C9—C8—N1	117.77 (14)
C1—C2—C3	119.60 (16)	C10—C9—C8	119.38 (17)
C1—C2—H2	120.2	C10—C9—H9	120.3
C3—C2—H2	120.2	C8—C9—H9	120.3
C4—C3—C2	120.09 (16)	C9—C10—C11	119.67 (17)
C4—C3—H3	120.0	C9—C10—H10	120.2
C2—C3—H3	120.0	C11—C10—H10	120.2
C3—C4—C5	120.47 (16)	C12—C11—C10	120.74 (16)
C3—C4—H4	119.8	C12—C11—H11	119.6
C5—C4—H4	119.8	C10—C11—H11	119.6
C4—C5—C6	121.05 (16)	C11—C12—C13	120.26 (17)
C4—C5—H5	119.5	C11—C12—H12	119.9
C6—C5—H5	119.5	C13—C12—H12	119.9
C5—C6—C1	116.82 (14)	C8—C13—C12	118.89 (16)
C5—C6—C7	123.27 (14)	C8—C13—H13	120.6
C1—C6—C7	119.85 (13)	C12—C13—H13	120.6
N1—C7—C6	126.52 (14)	O1—N1—C7	125.30 (14)
N1—C7—H7	116.7	O1—N1—C8	115.07 (12)
C6—C7—H7	116.7	C7—N1—C8	119.58 (13)
			
C6—C1—C2—C3	−0.8 (2)	N1—C8—C9—C10	178.21 (15)
Cl1—C1—C2—C3	178.84 (13)	C8—C9—C10—C11	−0.7 (3)
C1—C2—C3—C4	−0.8 (3)	C9—C10—C11—C12	0.0 (3)
C2—C3—C4—C5	1.7 (3)	C10—C11—C12—C13	0.5 (3)
C3—C4—C5—C6	−1.1 (3)	C9—C8—C13—C12	−0.5 (2)
C4—C5—C6—C1	−0.5 (2)	N1—C8—C13—C12	−177.63 (14)
C4—C5—C6—C7	−177.88 (16)	C11—C12—C13—C8	−0.3 (2)
C2—C1—C6—C5	1.4 (2)	C6—C7—N1—O1	−4.2 (3)
Cl1—C1—C6—C5	−178.22 (12)	C6—C7—N1—C8	178.20 (14)
C2—C1—C6—C7	178.92 (14)	C13—C8—N1—O1	133.68 (16)
Cl1—C1—C6—C7	−0.7 (2)	C9—C8—N1—O1	−43.6 (2)
C5—C6—C7—N1	−14.5 (2)	C13—C8—N1—C7	−48.5 (2)
C1—C6—C7—N1	168.21 (15)	C9—C8—N1—C7	134.23 (16)
C13—C8—C9—C10	0.9 (2)		

### Hydrogen-bond geometry (Å, °)

**Table:**

*D*—H···*A*	*D*—H	H···*A*	*D*···*A*	*D*—H···*A*
C5—H5···O1	0.93	2.26	2.857 (2)	121
C7—H7···Cl1	0.93	2.58	3.0206 (16)	110
